# Tick Activity, Host Range, and Tick-Borne Pathogen Prevalence in Mountain Habitats of the Western Carpathians, Poland

**DOI:** 10.3390/pathogens12091186

**Published:** 2023-09-21

**Authors:** Zbigniew Zając, Joanna Kulisz, Aneta Woźniak, Katarzyna Bartosik, Angélique Foucault-Simonin, Sara Moutailler, Alejandro Cabezas-Cruz

**Affiliations:** 1 Department of Biology and Parasitology, Medical University of Lublin, Radziwiłłowska 11, 20-080 Lublin, Poland; joanna.kulisz@umlub.pl (J.K.); aneta.wozniak@umlub.pl (A.W.); katarzyna.bartosik@umlub.pl (K.B.); 2 Anses, INRAE, Ecole Nationale Vétérinaire d’Alfort, UMR BIPAR, Laboratoire de Santé Animale, 94700 Maisons-Alfort, France; angelique.foucaultsimonin@anses.fr (A.F.-S.); sara.moutailler@anses.fr (S.M.)

**Keywords:** *Ixodes ricinus*, *Dermacentor reticulatus*, ticks, tick-borne pathogens

## Abstract

In mountainous regions, diverse ecosystems provide a habitat for numerous species of organisms. In this study, we focused on ixodid ticks and their presence in the Western Carpathians, Poland. Our objectives were to investigate the impact of environmental factors on tick occurrence and activity, the prevalence of vectored pathogens, and tick hosts, and their role as reservoir organisms for tick-borne pathogens (TBPs). To this end, we collected ticks from the vegetation and from animals (*Apodemus agrarius*, *A. flavicollis*, *Capreolus capreolus*, *Microtus* spp., *Myodes glareolus*, *Ovis aries*). In addition, we collected blood samples from rodents. The collected material underwent molecular analysis, utilizing the high-throughput microfluidic real-time PCR technique, to detect the presence of TBPs. Our findings confirmed the occurrence of only two species of ixodid ticks in the study area: the dominant *Ixodes ricinus*, and *Dermacentor reticulatus* with very limited abundance. Temperature significantly influenced tick activity, and the number of *I. ricinus* nymphs varied with altitude. We also observed a circadian pattern of questing activity in *I. ricinus* ticks. The main hosts for juvenile tick stages were *M. glareolus* and *A. agrarius*, while adult stages were frequently found on *C. capreolus*. *I. ricinus* ticks collected from the vegetation were often infected with *Rickettsia helvetica* (up to 35.71%), *Borrelia afzelii* (up to 28.57%), and *Ehrlichia* spp. (up to 9.52%). In contrast, juvenile stages frequently carried *Bartonella* spp. (up to 10.00%), *Mycoplasma* spp. (up to 16.67%) and *R. helvetica* (up to 16.67%). Moreover, we detected genetic material of *Mycoplasma* spp. (up to 100.00%), *Ehrlichia* spp. (up to 35.71%), *Bartonella* spp. (up to 25.00%), and *Borrelia* spp. (up to 6.25%) in rodent blood samples. The obtained results indicate *A. agrarius* and *M. glareolus* as reservoir animals for TBPs in the studied region.

## 1. Introduction

The Carpathians, the largest mountain range in Central Europe and second only to the Alps on the continent, harbor a significant portion of Europe’s biological diversity [1]. This diverse environment supports the occurrence of numerous species, including ticks, in the region and surrounding areas. One of the most important tick species in this area, from an epidemiological perspective, is *Ixodes ricinus*. This tick transmits spirochetes from the *Borrelia burgdorferi* sensu lato (s.l.) complex, which causes Lyme borreliosis (LB). Additionally, this tick species serves as the main vector of tick-borne encephalitis virus (TBEV), which is responsible for tick-borne encephalitis (TBE) [2,3]. Countries located in the Carpathian region, such as Poland, Slovakia, and Romania, report a high incidence of tick-borne diseases, mainly LB [4,5].

Other prevalent tick species in Central Europe are *Dermacentor marginatus* and *D. reticulatus*, whose populations have undergone substantial changes (expansion/increase) in recent years. This is most probably due to climate warming and alterations in habitat structure, affecting the availability of tick hosts [6,7]. *Dermacentor* ticks have frequently been shown to transmit *Rickettsia* spp., responsible for tick-borne fever group rickettsioses in humans [8,9]. *D. reticulatus* has also been confirmed as a competent vector for TBEV and *Babesia canis*, which causes canine babesiosis, making it crucial from a veterinary medicine point of view [10,11]. In addition to the mentioned ticks, *Rhipicephalus sanguineus*, *R. bursa*, *Hyalomma marginatum*, and *Haemaphysalis concinna* ticks, known vectors of pathogens such as *Babesia* spp. and *Rickettsia* spp., have been reported in areas adjacent to the Carpathians [12,13].

So far, in Poland, 19 species of ixodid ticks have been found (excluding imported species), with *I. ricinus* having the largest population size across the country, while *D. reticulatus* does not occur in the central part of Poland [14,15]. Little is known about the distribution of ticks in the southern mountainous region of the country. In this area, ticks play a vital role in various ecological processes, including disease transmission, and understanding their distribution, activity, and host spectrum is crucial. Furthermore, these ticks may serve as vectors for tick-borne pathogens (TBPs), which pose a significant threat to both wildlife and human health. Therefore, investigating the distribution, activity, host spectrum, and the presence of TBPs in ticks within this region is of paramount importance. Some of the studies have confirmed the presence of *I. ricinus* and an insular distribution of *I. hexagonus*, *I. arboricola,* and *I. trianguliceps* in this area, with *Anaplasma phagocytophilum* and *B. microti* as the most prevalent TBPs [14,16,17]. In turn, a broader tick species spectrum (with a compact occurrence range) and a relatively large number of ticks are observed in countries bordering Poland along the Carpathians [13,18,19].

The focus of our study was on the Western Carpathians, where we hypothesized the co-occurrence of ixodid tick species. We aimed to investigate the impact of environmental factors on tick occurrence and activity, as well as the prevalence of vectored pathogens. Additionally, we explored the potential tick hosts and the role of rodents as reservoir animals for TBPs in this region.

## 2. Materials and Methods

All methods were carried out in accordance with the relevant guidelines and regulations. The studies on animals were carried out with the consent of the Local Ethics Committee at the University of Life Sciences in Lublin (permit no. 97/2022) and was carried out in compliance with the ARRIVE guidelines (Animal Research: Reporting of in vivo Experiments). The research was designed following the 3R principle (Replacement, Reduction, and Refinement) in order to limit the number of animals used to the minimum necessary for the credibility of the results and ensuring sufficient amounts of data for statistical analysis.

### 2.1. Study Area

**Figure 1:**
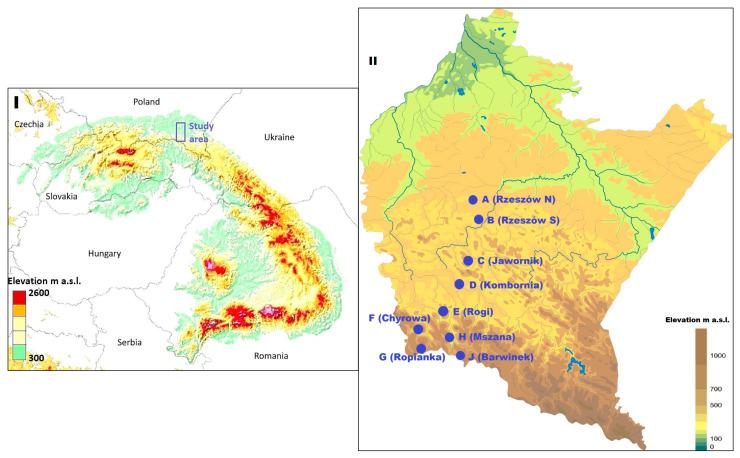
Overview map of the location of the Carpathians in the context of Europe (**I**) and the study sites in the context of southeastern Poland (**II**). This figure was generated using an online tool https://opentopomap.org, accessed on 1 June 2023 and edited in GIMP 2.20.32 software (GIMP Development Team, https://www.gimp.org, accessed on 1 June 2023).

The Carpathians are a mountain chain stretching with its foothills across the Central European countries, i.e., Austria, the Czech Republic, Poland, Slovakia, Ukraine, Hungary, Romania, and Serbia (Figure 1). In terms of its physico-geographical division, this mountain range is divided into the Western, Eastern, and Southern Carpathians [20].

The field study was conducted in the area of the Western Carpathians (the Central Beskids subregion) and their foothills. The mountain region has a temperate climate with features of continentalism, an average annual air temperature of 6.3 °C (1950–2010), with an increase to 8.5 °C recorded in the last decade, and an average annual rainfall of 885 mm. On average, the maximum air temperature does not exceed 0.0 °C for 58 days per year, and the snow cover persists for 93 days. The foothills have a milder climate, with an average temperature of 8.9 °C and an average annual rainfall of 771 mm. The maximum air temperature does not exceed 0.0 °C for 40 days per year, and the snow cover persists for 73 days [21].

The highest peak in the Polish part of the Central Beskids range (Lackowa Mountain) reaches 997 m a.s.l. There are only two vegetation floors here, i.e., the foothill zone with the dominance of Tilio Carpinetum association forests and the subalpine zone dominated by vegetation from the Dentario glandulosae-Fagetum association and fir trees (*Abies alba*) [22].

### 2.2. Occurrence and Seasonal Activity of Ticks

The field study of the occurrence and seasonal activity of ticks was carried out from June 2022 to July 2023, with the exception of winter months (November–March). The ticks were collected at monthly intervals, excluding the winter period with snow cover. The collection sites were established based on tick habitat preferences [8,16], taking into account the peculiarities of the locally dominating vegetation. In the area of the foothills, there were five tick collection sites, i.e., sites A (Rzeszów N) and E (Rogi), located in a meadow with progressive ecological succession, as well as sites B (Rzeszów S), C (Jawornik), and D (Kombornia), in a mixed forest. In turn, four sites were selected in the mountain area, i.e., sites F (Chyrowa) and H (Mszana), located in an agriculturally unused meadow with progressive ecological succession, as well as sites G (Ropianka) and J (Barwinek), located in a mixed forest (Figure 1). The dominant vegetation in the meadow habitats was from the class Molinio-Arrhenatheretea and birches (*Betula* spp.), while Tilio Carpinetum (in the foothols) and Dentario glandulosae-Fagetum (in the mountains) dominated in the forest habitats.

In order to investigate the seasonal activity of ticks, the specimens were collected from the vegetation with the flagging method by sweeping the plants with a 1 m^2^ white flannel cloth. At each site, ticks were collected in an approximately 100 m long transect for 30 min. Tick specimens were placed in a 50 cm^3^ plastic container. Additionally, a Data Logger R6030 device (R6030, Reed Instruments, Wilmington, NC, USA) was used each time during the field study to measure the current air temperature and humidity at the vegetation level.

Next, the collected ticks were transported to the laboratory, where their developmental stage, sex, and species were identified with the use of a Zeiss STEMI DV4 stereoscopic microscope (Carl Zeiss Light Microscopy, Göttingen, Germany) and a taxonomic identification key [13]. Then, the specimens were kept frozen at −80 °C (Arctico, Esbjerg, Denmark) until DNA extraction.

### 2.3. Circadian Tick Activity

The circadian activity of the ticks was analyzed during the *I. ricinus* spring activity in April–June [4] in different ecological types of habitat: a meadow biotope (site F, Chyrowa) and a forest biotope (site G, Ropianka). The investigations were carried out in newly designated transects (100 m length) in the same habitats in which the seasonal activity of ticks was analyzed.

The circadian activity of the ticks was monitored over the turn of two consecutive days each time. Ticks were collected using the flagging method and, after the identification of the developmental stage and the species, released at exactly the same collection site. The activity was monitored continuously in 4 h intervals, i.e., at 18:00, 22:00, 2:00, 6:00, 10:00, and 14:00.

### 2.4. Host Range

From August 2022 to July 2023, ticks were collected from animals (mammals) that are commonly regarded as their hosts in order to determine the spectrum of tick hosts in the habitats of the Western Carpathians [13,14,16]. In cooperation with a local breeder, a flock of 75 Corriedale sheep grazing on an approximately 2 ha pasture was monitored. Once a week, the animals’ bodies were inspected for the presence of ticks as part of other treatments to maintain their welfare. Ticks were also collected from wild game, i.e., roe deer (*C. capreolus*) culled as part of a program for the control of the size of the local population of this species. After removal from the host body, ticks were placed in a plastic container and preserved in a 70% ethanol solution until further analysis.

Additionally, small rodents were captured at sites F (meadow biotope) and G (forest biotope) between October 2022 and June 2023. To this end, 40 live-catch traps (Model S1, R-Max, Mrągowo, Poland) were placed at distances of 2 m at both sites. A mixture of sunflower seeds, oats, and rye was used as bait. Additionally, larvae of *Zophobas morio* insects were placed in the traps, taking into account the potential accidental capture of carnivorous rodents. In accordance with the methodology proposed by Skuratowicz [23], the traps were placed at the sites late in the evening and inspected every 4 h on the subsequent day. Captured rodents were identified to the species/genus [24], and their bodies were inspected for the presence of ticks, which were removed with metal tweezers and transferred into a container with 70% ethanol.

In this study, the prevalence of ticks was calculated on the basis of all trapped rodents, including both tick-infested and non-infested animals. The mean intensity of infestation, on the other hand, was calculated exclusively on the basis of tick-infested rodents, representing the average number of ticks per animal that was found to be infested.

### 2.5. Blood Collection Protocol

In the next step, blood samples were collected from the most abundant rodent species in a given habitat, i.e., *Apodemus agrarius* in the meadow habitat (F) and *Myodes glareolus* in the forest biotope (G). In total, 30 animals were included in the study. To collect blood samples (up to 10 µL), the skin was disinfected and punctured with a sterile needle, and then blood was collected using capillary action. The blood samples were transferred to 1.5 mL Eppendorf tubes.

Subsequently (throughout the field study), the blood samples were flooded with 70% ethanol after coagulation. In the laboratory, ethanol was removed, the clot surface was washed twice with distilled water, and the samples were frozen at −20 °C until further molecular analysis.

### 2.6. Molecular Analysis

The molecular analyses for detecting the presence of TBPs involved 168 randomly chosen adult *I. ricinus* ticks collected in mountain and foothill habitats representing different ecological types (equal numbers of samples). Additionally, genetic material was isolated from juvenile ticks collected both from vegetation (72 *I. ricinus* nymphs) and from rodent hosts (36 larvae and 114 nymphs of *I. ricinus* and 18 *D. reticulatus* nymphs). Due to the low degree of blood ingestion, the nymphs and larvae were pooled into groups of 6 specimens. In addition, 30 blood samples (16 from *M. glareolus* and 14 from *A. agrarius*) were analyzed for the presence of the genetic material of TBPs.

#### 2.6.1. DNA Extraction

In the first stage, ticks collected during the field study were rinsed with distilled water, dried, and cut into smaller fragments using a sterile scalpel. Tick DNA was extracted with the column method using the Genomic Mini AX Tissue kit (A&A Biotechnology, Gdynia, Poland), whereas the Blood Mini kit (A&A Biotechnology, Gdynia, Poland) was used to isolate DNA from the blood samples following the manufacturer’s instructions. The concentration of DNA (15–300 ng/µL in a total volume of 35 µL) was measured using a NanoDrop 2000 spectrophotometer (Thermo Scientific, Waltham, MA, USA) at a 260/280 nm wavelength. Next, the samples were stored at −20 °C until further processing.

#### 2.6.2. DNA Preamplification

The PreAmp Master Mix kit (Fluidigm, San Francisco, CA, USA) was used for DNA preamplification. The procedure was conducted following the manufacturer’s protocol. All primer pairs targeting TBPs [25,26] were pooled, combining equal volumes with a final concentration of 0.2 µM each in a final volume of 5 μL containing 1 μL Perfecta Preamp 5×, 1.25 μL pooled primer mix, 1.5 µL distilled water, and 1.25 μL DNA. The thermocycling included one cycle at 95 °C for 2 min, 14 cycles at 95 °C for 15 s, and 4 min at 60 °C. After that, the products were diluted 1:10 in Milli-Q ultrapure water (45 μL) and stored at −20 °C until further analysis.

#### 2.6.3. Microfluidic Real-Time PCR for High-Throughput Detection of Microorganisms

The BioMark™ real-time PCR system (Fluidigm, San Francisco, CA, USA) was run for detection of pathogens, including *Borrelia* spp., *B. burgdorferi* s.s., *B. garinii*, *B. afzelii*, *B. valaisiana*, *B. lusitaniae*, *B. spielmanii*, *B. bissettii*, *B. miyamotoi*, *Anaplasma marginale*, *A. platys*, *A. phagocytophilum*, *A. ovis*, *A. centrale*, *A. bovis*, *Ehrlichia canis*, *N. mikurensis*, *Rickettsia* spp., *R. slovaca*, *R. helvetica*, *Bartonella henselae*, *Francisella tularensis*, *Mycoplasma* spp., *Babesia microti*, *B. canis*, *B. ovis*, *B. bovis*, *B. caballi*, *B. venatorum*, and *B. divergens* [25,26].

Real-time PCR reactions were performed using 6-carboxyfluorescein (FAM)-labeled and black hole quencher (BHQ1)-labeled TaqMan probes with TaqMan Gene expression master mix according to the manufacturer’s protocol (Applied Biosystems, Les Ulis, France). The reaction consisted of the following steps: 2 min at 50 °C, 10 min at 95 °C, followed by 40 cycles of two-step amplification of 15 s at 95 °C, and 1 min at 60 °C.

In the real-time PCR reaction, we applied primers identical to those used in a previously published paper [25,26]. Primers targeting *I. ricinus* and *D. reticulatus* DNA were used to confirm tick species (previously described based on morphological features). *Escherichia coli* DNA and *Escherichia*-specific primers were used as positive controls [25]. Ultra-pure water was used for a negative control. The results were analyzed using Fluidigm Real-time PCR Analysis Software v2.1 to calculate crossing threshold (Ct) values.

#### 2.6.4. Validation of Microfluidic Real-Time PCR and Phylogenetic Analysis

To validate the obtained results, randomly selected positive samples underwent conventional PCR assay using different primers of the BioMark real-time PCR system as described previously [25,26]. In addition, selected *Borrelia* positive samples (targeting *flaB* gene) were sequenced by Eurofins MWG Operon (Ebersberg, Germany) to confirm the genospecies of the pathogen. Obtained sequences were analyzed using BioEdit software v7.0 (Ibis Biosciences, Carlsbad, Germany) and submitted to the GenBank database under the following accession numbers: OR544356-OR544359, OR544362.

Obtained sequences were analyzed using the Basic Local Alignment Search Tool (BLAST; https://blast.ncbi.nlm.nih.gov/Blast.cgi, accessed on 1 September 2023). In the next step, sequences showing similarity were aligned using the MUSCLE algorithm in MEGA 11 (https://www.megasoftware.net/, accessed on 1 September 2023). Based on the lowest Bayesian Information Criterion and corrected Akaike Information Criterion, the Kimura 2-parameter (K2) model was used to build the tree using the Maximum Likelihood (ML) method. The reliability of internal branches was assessed using the bootstrapping method with 1000 replicates.

### 2.7. Statistical Analysis

#### 2.7.1. Prevalence of TBPs in Pooled Samples

To estimate the prevalence of TBPs in pooled samples (juvenile ticks collected from rodents), the minimum infection rate (*MIR*) was calculated using the following formula:

$$MIR=\frac{Pp}{TP\times TNP\;}\times 100\%$$

where: *MIR*—minimum infection rate; *Pp*—number of positive pools; *TP*—number of tested pools; *TNP*—pool size.

#### 2.7.2. Analysis of the Significance of Results

The normality of the obtained data was checked using the Shapiro–Wilk test. The effect of air temperature and relative humidity on tick activity was examined using Spearman’s rho correlation. Similarly, this test was applied to analyze the relationship between altitude and the number of active ticks. The non-parametric ANOVA test was used to check the significance of differences in the number of active ticks between habitats, while the repeated measures ANOVA test was used to analyze the differences in the number of active ticks in relation to the time of day/night. The chi-square test was used to analyze the prevalence of TBPs. Similarly, this test was also used to examine the significance of tick infestation between animals.

The significance level was set at *p* < 0.05. Statistical calculations were performed using the STATISTICA 13.3 PL statistical package (StatSoft, TIBCO Software Inc., Palo Alto, CA, USA) and GraphPad 8.4 (GraphPad Software Inc., La Jolla, CA, USA).

## 3. Results

### 3.1. Occurrence and Seasonal Activity of Ticks

**Figure 2:**
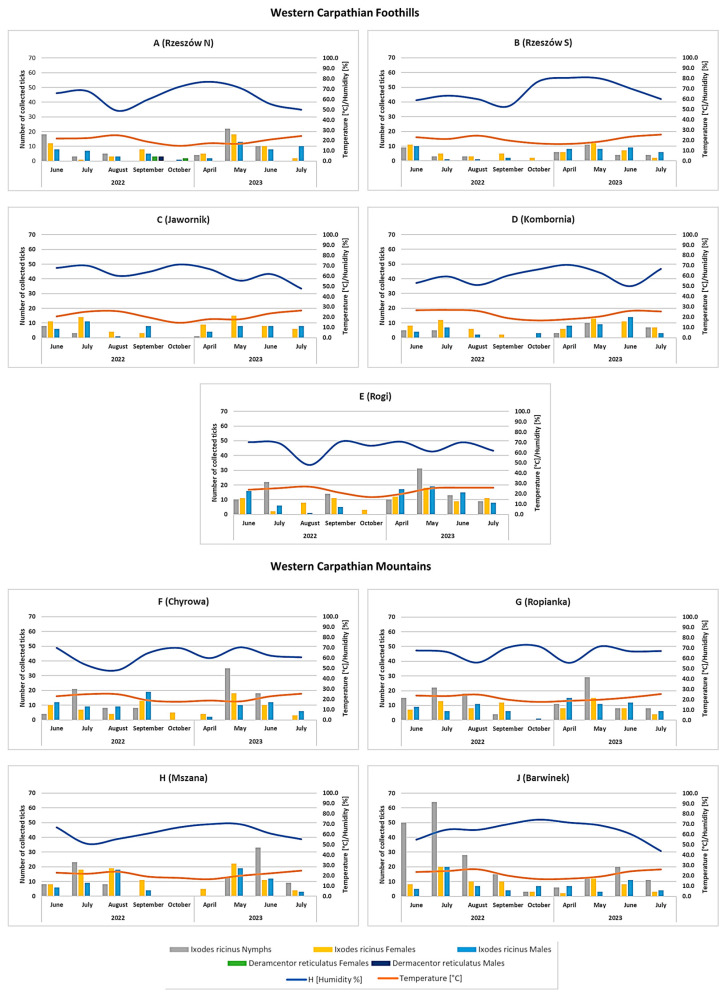
Occurrence and seasonal activity of ixodid ticks in the studied region.

During the study period, a total of 2016 ticks were collected from the vegetation. *I. ricinus* was the dominant species at all sites, with 2008 specimens collected, including 765 nymphs, 655 females, and 588 males (Figure 2, Table S1). In contrast, *D. reticulatus* ticks were only collected from vegetation in site A (Rzeszów N) situated in the foothills, where five females and three males were collected (Figure 2). Both in the foothills and in the mountains, the peak activity of *I. ricinus* ticks was observed in June–July, when up to 64 nymphs were collected during one collection event (site J Barwinek) (Figure 2). The ticks were active in the temperature range of 14.5–27 °C and at 43.9–80.5% relative humidity (Figure 2).

A weak but statistically significant positive correlation was found between the thermal conditions and the number of active *I. ricinus* ticks (r_s_ = 0.2705, *p* = 0.0139). However, no significant relationship was observed with relative air humidity (r_s_ = 0.030, *p* = 0.7854).

When comparing habitats, a greater number of ticks was collected from habitats located in the mountain area (sites F–J) (1130 specimens in total) than in the foothills (878 ticks; sites A–E) (Figure 2). Along with altitude gradient, there was a positive, statistically significant correlation between the number of active nymphs of *I. ricinus* (r = 0.2709, *p* = 0.0098), but no significant relationships were found in females (r = 0.1547, *p* = 0.1471) or males (r = 0.1438, *p* = 0.1764).

### 3.2. Circadian Activity of Ixodes ricinus Ticks

**Figure 3:**
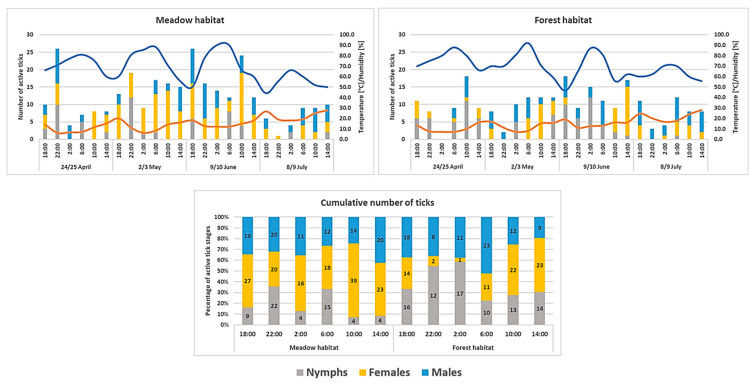
Circadian activity of *Ixodes ricinus* ticks in the Western Carpathian region.

The present study revealed a circadian pattern of *I. ricinus* questing activity, with its rhythm and intensity varying depending on the time of day and the development stage. Circadian activity of *I. ricinus* ticks was observed in temperature range 6.1–28.0 °C and relative humidity 44.1–92.0% (Figure 3).

In the forest habitat, the majority of *I. ricinus* nymphs were active at night, specifically between 22:00 and 2:00. However, their activity did not significantly differ from that of other nymphs during the daytime (F = 0.2500, *p* = 0.6667). During the daytime (10:00–14:00), the activity of nymphs in the forest habitat was higher than that of the nymphs collected in the meadow habitat. Additionally, a statistically significant difference was found in the number of active *I. ricinus* females depending on the time (night/day) in the forest habitat (F = 25.0000, *p* = 0.00377), but not in the meadow habitat (F = 2.1889, *p* = 0.2212). In the meadow habitat, the most active *I. ricinus* females were observed at 2:00–10:00 (Figure 3). There were statistically significant differences in the circadian activity of *I. ricinus* females collected from the forest and meadow habitats (F = 12.2981, *p* = 0.0018).

### 3.3. Host Range

Tick infestation was confirmed in all the animal species analyzed. *I. ricinus* ticks were collected from wild rodents *M. glareolus*, *Microtus* spp., *A. agrarius*, medium-sized game *C. capreolus*, and farmed sheep *O. aries*. In turn, *D. reticulatus* ticks were found to infest *A. flavicollis* (the only tick species identified in this host), *Microtus* spp., and roe deer *C. capreolus* (Table 1).

The prevalence of ticks was notably high in *C. capreolus*, reaching 100.00%. Additionally, *M. glareolus* (88.9%) and *A. agrarius* (58%) reached considerable tick infestation levels. The rodents captured in the forest habitat exhibited a statistically significant higher degree of tick infestation than the animals from the meadow ecosystem (χ^2^ = 10.05, *p* = 0.0015). The highest mean infestation intensity was observed in *C. capreolus* (7.5) and *Microtus* spp. (7.0) (Table 1).

### 3.4. Prevalence of TBPs in Questing Ixodes ricinus Ticks

#### 3.4.1. Prevalence and Phylogeny of Spirochetes of the Borreliaceae Family

**Figure 4:**
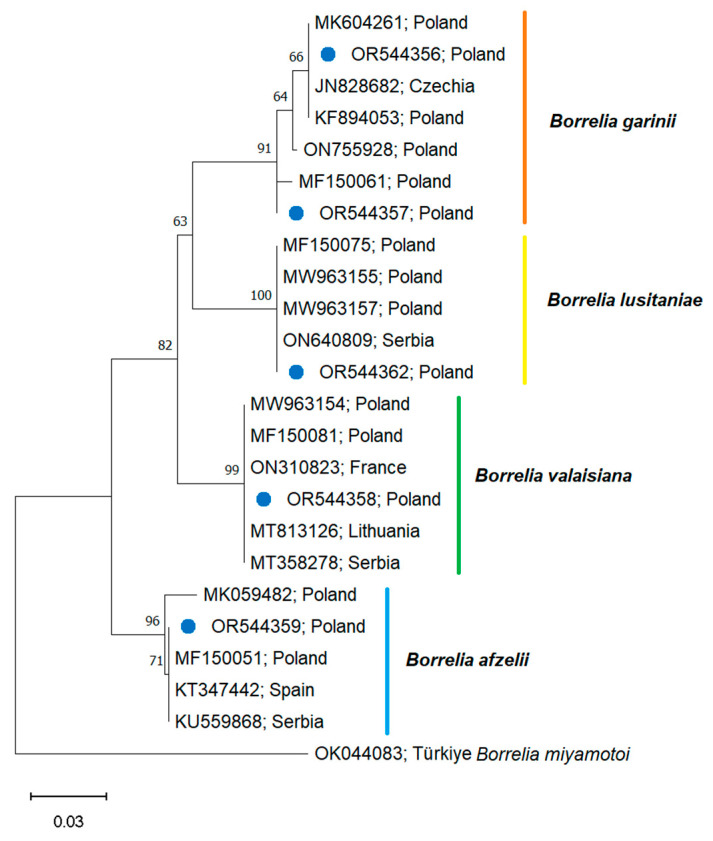
Phylogeny of Borreliaceae family based on *flaB* gene. The evolutionary history was inferred by using the Maximum Likelihood method and the Kimura 2-parameter model. The analysis contains sequences identified in the current study (marked with blue dot) and GenBank sequences. Accession numbers of sequences and country of origin are given. Bootstrap values are represented as percentage of internal branches (1000 replicates), and values lower than 60 are hidden. The tree is drawn to scale, with branch lengths measured in the number of substitutions per site. *Borrelia miyamotoi* sequence OK044083 was used to root the tree.

The presence of the genetic material of Borreliaceae spirochetes, i.e., *B. burgdorferi* s.s., *B. afzelii*, *B. garinii*, *B. valaisiana*, *B. lusitaniae*, *B. spielmanii* (responsible for human LB), and *B. miyamotoi* (a causative agent of tick-borne relapsing fever (TBRF), was detected in *I. ricinus* ticks collected in the study area (Figure 4, Table 2).

The most common *Borrelia* genospecies detected in adult *I. ricinus* ticks were *B. afzelii* (up to 28.57%) and *B. garinii* (up to 21.42%). In the mountain area, a significantly higher prevalence of infections with Borreliaceae spirochetes was observed in the meadow habitat than in the forests (χ^2^ = 9.4169, *p* = 0.0021). *I. ricinus* nymphs were infected with *B. afzelii* and *B. garinii* (Table 2).

Phylogenetic analysis showed that the *Borrelia* sequences obtained in the current study clustered together with others previously reported from Poland and/or from the region.

#### 3.4.2. Prevalence of Pathogens of the Anaplasmataceae Family

Infection with Anaplasmataceae pathogens, namely *A. phagocytophilum* and *Ehrlichia* spp., was detected only in *I. ricinus* adults (Table 2). Only one female (2.38%) from the forest habitat in the mountain area was infected with *A. phagocytophilum*, while *Ehrlichia* spp. were detected in ticks in each of the examined sites, and the prevalence of infections with this pathogen reached up to 9.52% (Table 2). There were no significant differences in the prevalence of *Ehrlichia* spp. between the habitats in the mountains (χ^2^ = 0.3546, *p* = 0.5515) and between the mountains and the foothills (χ^2^ = 0.0827, *p* = 0.7742).

#### 3.4.3. Prevalence of Bacteria of the Rickettsiaceae Family

The genetic material of *Rickettsia* spp. (up to 7.14%) and *R. helvetica* (up to 35.71%) was detected in adult *I. ricinus* ticks from each of the sites (Table 2). There were no significant differences in the prevalence of *R. helvetica* in ticks collected in the mountain habitats (χ^2^ = 0.1569, *p* = 0.6921), whereas significantly higher numbers of *I. ricinus* ticks were infected with this pathogen in the foothill habitats than in the mountain localities (χ^2^ = 3.7400, *p* = 0.04942).

#### 3.4.4. Prevalence of the Protozoan Parasite *Babesia venatorum*

*B. venatorum* infection was confirmed in two *I. ricinus* females: one collected in the forest habitat in the mountains and the other collected in the foothills (2.38%) (Table 2).

#### 3.4.5. Coinfection with TBPs in Questing *Ixodes ricinus* Ticks

A prevalence of co-infections with tick-borne pathogens ranged up to 7.14% was detected in *I. ricinus* adults collected in the study area (Table S2). The co-infections involved up to two pathogens, with polymicrobial infections mainly caused by different genospecies of *Borrelia* spp. and *R. helvetica* or *Ehrlichia* spp. (Table S2).

### 3.5. Rodents as a Reservoir of TBPs in Nature

#### 3.5.1. Prevalence of TBPs in Juvenile Ticks Collected from Rodents

The minimum infection rate (*MIR*) was calculated to assess the prevalence of TBPs in the juvenile tick stages (pooled samples). The juvenile stages of *I. ricinus* collected from the rodents were characterized by a lower prevalence of TBPs than the adults of this species collected from the vegetation (Table 2 and Table 3). The most frequent pathogens were *Mycoplasma* spp. (up to 16.67% *MIR*), *Rickettsia* spp. (up to 16.67% *MIR*), and *Bartonella* spp. (up to 10.00% *MIR*). The *MIR* value in the case of Borreliaceae spirochetes was estimated at up to 3.33% (Table 3).

#### 3.5.2. Prevalence of TBPs in Rodent Blood Samples

The genetic material of *Borrelia* spp., *Ehrlichia* spp., *Bartonella* spp., and *Mycoplasma* spp. was detected in the rodent blood samples. (Table 4). *Mycoplasma* spp. was the most frequently detected pathogen in both of these rodent species. A high prevalence of *Bartonella* spp. was observed in *M. glareolus*, whereas *Ehrlichia* spp. bacteria were detected in *A. agrarius* only from the meadow habitat. Noteworthy is the low prevalence of *Borrelia* spp. spirochetes (Table 4). A statistically significant relationship was found between the overall prevalence of the detected pathogens and the rodent host/habitat (χ^2^ = 46.9136, *p* < 0.0001).

Polymicrobial infections were detected in the blood samples as well. The *M. glareolus* specimens were found to be coinfected with up to two pathogens, i.e., *Borrelia* spp. + *Mycoplasma* spp. (6.25% of the examined animals) and *Bartonella* spp. + *Mycoplasma* spp. (in 18.75%). A higher prevalence of co-infections was observed in *A. agrarius*, which were co-infected with three pathogens, i.e., *Ehrlichia* spp. + *Bartonella* spp. + *Mycoplasma* spp. (in 7.14% of the samples) (Table 4). These differences were statistically significant (χ^2^ = 13.3333, *p* = 0.0002).

## 4. Discussion

The results of the present study confirmed the occurrence of only two species of ixodid ticks in the Western Carpathians and their foothills (south-eastern Poland), i.e., the dominant *I. ricinus* species and the less frequent *D. reticulatus* (Figure 2).

*I. ricinus* is the most widespread tick species in Europe, and its compact occurrence range covers the entire territory of Poland and neighboring countries [13]. This was confirmed by the results of our study, as we found *I. ricinus* ticks at all of the sites in both the foothills and the mountains and in both (meadow and forest) habitat types (Figure 2, Table S1). In contrast, *D. reticulatus* ticks are characterized by an insular occurrence range on both continental and local scales [6,27]. In Poland, *D. reticulatus* is the most abundant tick species in the eastern part of the country [28], but its population size and occurrence range in the central and western parts are increasing dynamically [15]. Therefore, our results showing the presence of *D. reticulatus* adults on vegetation only in the northernmost site, A (Rzeszów N) (Figure 2), allow the assumption that the area of the Western Carpathian foothills is currently a border of the geographical range of *D. reticulatus* in this region. Notably, we detected the presence of nymphs of this species feeding on small rodents from the mountain habitats and collected adults infesting medium-sized game (*C. capreolus*) (Table 1). However, we believe that the limited numbers of these ticks collected in this region may indicate accidental introduction of *D. reticulatus* rather than their regular occurrence in this area. The hypothesis of accidental import may also be supported by the fact that juvenile *D. reticulatus* stages were collected both from *Microtus* rodents (meadow habitat), which are regarded as the main spectrum of hosts of this tick species [29], and from *A. flavicollis* (forest habitat), which may be potential hosts of *D. reticulatus*, but their infestations by this species are less frequent due to the different habitat preferences of the host (*A. flavicollis*) and the tick (*D. reticulatus*) [11]. Nevertheless, this phenomenon may contribute to future changes in the occurrence range of this tick species in the Western Carpathians.

Our results confirm the wide ecological tolerance of *I. ricinus* in relation to the habitats occupied by this species. We collected *I. ricinus* ticks in the forest and meadow habitats located both in the foothills and in the mountains (Figure 2). As reported in the literature, *I. ricinus* ticks prefer primarily deciduous or mixed forest habitats, but they can also be found in pastures, moorlands, and urban parks [4,30,31,32,33]. The thermal and humidity conditions are the key determinants of the occurrence and activity of *I. ricinus* ticks [34]. Previously published studies on the distribution of *I. ricinus* in Poland showed that these ticks were active in the temperature range of 7.5–28.8 °C and the optimal humidity level of 70% [4,35]. Similarly, the present study showed a significant positive correlation between the temperature and the number of active ticks. Interestingly, there was no relationship with the humidity conditions. The relative humidity in the habitats ranged from 43.9% to 80.5%, with an average value of 63.5% (Figure 2), which ensured optimal conditions for the activity of these ticks, including juvenile stages, which are extremely vulnerable to water loss [36,37,38].

The sites analyzed in this study were located at 191–529 m a.s.l. (Table S1). We found a significant difference in the number of active *I. ricinus* nymphs along the altitude gradient, i.e., they were more numerous in sites located at higher altitudes (max. 64 specimens, site J Barwinek, 470 m.a.sl.) (Figure 2, Table S1). We believe that this is related to the specificity of the local microclimatic conditions and vegetation. This site was located in a deciduous forest with a dense layer of herbaceous plants and grasses. Studies conducted in Europe confirm the occurrence of *I. ricinus* ticks in mountain ecosystems, with simultaneous indication of changes in the upper occurrence range of this species noted over the last few decades [39,40,41]. As reported by Rosický (1954) [42] and Černý (1965) [43], *I. ricinus* ticks reach an altitude of approximately 700–800 m a.s.l., while Garcia-Vozmediano et al. (2020) [40] indicated the upper boundary as being approximately 1800 m a.s.l.

In the Western Carpathians and the foothill area, the peak activity of *I. ricinus* ticks is recorded in June–July, i.e., about a month later than in eastern Poland (May–June) [4]. We believe that this is associated with the different climatic conditions prevailing in the analyzed region with prolonged frosty weather (40–58 days), lower average annual air temperature (8.5–8.9 °C), and consequently a shorter growing season (170 days) [21]. The peak *I. ricinus* activity in the mild marine climate zone in Ireland has previously been recorded as being at the end of March and the beginning of April [44], whereas the peak activity of this species in southern Germany begins in May [45].

Our study confirmed that *I. ricinus* ticks have a circadian rhythm of questing activity, and its intensity varies depending on the time of day/night and the tick developmental stage. The *I. ricinus* nymphs from the forest habitat exhibited high activity at night, which was also higher during the day than the activity of this tick stage in the meadow habitat (Figure 3). We believe that this was associated with the more favorable water balance maintained in the forest habitat; it prolonged the activity of the juveniles, which are characterized by high vulnerability to water loss [46]. It has also been reported that ticks that are active at night are less likely to be desiccated [47,48]. Interesting but not fully elucidated is the effect of the infection with some pathogens, e.g., *A. phagocytophilum*, on the expression of genes involved in the regulation of circadian rhythms in ticks [49].

We detected tick infestations in all of the analyzed animal species. The highest prevalence and mean intensity of tick infestations was observed in the *C. capreolus* roe deer (Table 1). Game animals, e.g., *C. capreolus* (especially the forest ecotype), are one of the most important hosts of *I. ricinus* adult stages [50]. Noteworthy, *D. reticulatus* males feeding on roe deer were found in the present study, as well (Table 1). With their habitat preferences and the circadian rhythm of feeding and resting phases, *C. capreolus* roe deer are easily accessible and preferable tick hosts. Additionally, previous studies have shown a correlation between the density of local populations of *C. capreolus* and the incidence of tick-borne diseases in humans [51,52,53].

In the analysis of the tick host range, we also monitored another group of medium-sized animals, i.e., a flock of Corriedale sheep (*O. aries*), and confirmed the presence of *I. ricinus* females feeding on the sheep. However, these animals exhibited the lowest prevalence (6.7%) and intensity of tick invasion (3.4) among all the analyzed groups of animals (Table 1). Although sheep are regarded as potential *I. ricinus* hosts [54], Steigedal et al. [55] suggested that sheep alter vegetation and thus reduce tick survival, thereby limiting the risk of tick infestation of other grazing animals.

A high prevalence of *I. ricinus* juvenile stages was observed in *M. glareolus* (88.9%), *A. agrarius* (58.3%), and *Microtus* spp. (50.0%). The intensity of tick invasion in the animals from the forest habitat was significantly higher, which may have been related to the favorable humidity conditions prevailing in this biotope (Table 1). These results are consistent with previous reports indicating these rodent species as the main hosts of the juvenile stages of ixodid ticks [56,57,58]. Therefore, the present results allow the conclusion that the host range of *I. ricinus* juvenile stages in the Western Carpathians corresponds to the host preferences of populations of this species from other regions.

The present results show that *I. ricinus* adult stages (especially females) are an important vector and reservoir of TBPs in the analyzed region. The ticks were most often infected with Borreliaceae spirochetes (up to 28.75%). We also confirmed a broad spectrum of *Borrelia* genospecies in the *I. ricinus* specimens (Table 2). The similar spectrum of *Borrelia* genospecies and a similar level of prevalence were confirmed in our earlier studies conducted in eastern Poland (up to 25.00%) [8,59]. *B. afzelii* and *B. garinii* are the dominant genospecies in the Western Carpathian region (Table 2), similar to the trends observed in other Central European countries [60]. In addition, our results confirm the genetic similarity of *Borrelia* genospecies isolated from *I. ricinus* ticks in the study region and those reported from other countries and regions (Figure 4).

*I. ricinus* adults were found to play an important role as a vector of *R. helvetica* in the study area. This bacterium was detected in up to 35.71% of ticks collected in the foothills. A high level of infection with this pathogen has also been reported in other regions [61,62], which suggests that these ticks can be regarded as a major reservoir of *R. helvetica*, and vertebrate hosts play important roles in the further geographical dispersion of rickettsiae [63].

In the analyzed region, we also confirmed the presence of bacteria from the Anaplasmataceae family, i.e., *A. phagocytophilum* and *Ehrlichia* spp., in *I. ricinus* (Table 2). Up to 9.52% of the ticks were infected with *Ehrlichia* spp. (Table 2). A high prevalence of infections with this pathogen, reaching up to 35.7%, has also been reported in other European countries. *A. phagocytophilum* infection was confirmed in only one *I. ricinus* female (2.38%) from the mountain habitat (Table 2). Infections with this pathogen in ticks have been reported in most European countries, with a prevalence in the range of 0.5–34% [64,65]. In other studies, the type of habitat and the availability of potential hosts have been shown to be the main factors of *A. phagocytophilum* infections in ticks [33]. In comparison with the present study, of particular interest is the negative correlation of the *A. phagocytophilum* prevalence with altitude reported by other authors [66]. In contrast, the prevalence of spotted fever group (SFG) rickettsiae increased with altitude, and *R. helvetica* was the only TBP detected above 1800 m a.s.l. in the Italian Alps [40].

The results of our study revealed a high degree of TBP co-infections in adult *I. ricinus* ticks (Table S2). Co-infections are common phenomena; however, the species composition and prevalence of pathogens involved in co-infections may vary depending on the tick host species, the ecological type of tick habitats, and the geographical region [67,68]. Co-infections in *D. reticulatus* are often caused by *R. raoulti*, *Francisella*-like endosymbiont (FLE), *Bartonella* spp., and *Borrelia* spp. [8], whereas co-occurrence of Borreliaceae genospecies, *R. helvetica*, *Neoehrlichia mikurensis*, and *A. phagocytophilum* is most frequently diagnosed in *I. ricinus* [69,70].

Rodents are the main hosts for juvenile forms of ixodid ticks [11,71]. As shown in the present study, *I. ricinus* larvae and nymphs from the habitats of the Western Carpathians were most frequently infected with *R. helvetica* (*MIR* up to 16.67%) and *Mycoplasma* spp. (*MIR* up to 16.67%), while the value of *MIR* of *Borrelia* spirochetes was 3.33% (Table 3). In turn, the results of other studies conducted in Switzerland demonstrated prevalences of *R. helvetica* and *Babesia* spp. in juvenile *I. ricinus* ticks of 7.2% and 2.4%, respectively, whereas no *A. phagocytophilum* infection was detected [72].

Wild-living rodents play an important role as TBP reservoir animals in nature [73]. Our study showed the presence of the genetic material of *Borrelia* spp., *Ehrlichia* spp., *Bartonella* spp., and *Mycoplasma* spp. in the rodent blood samples, with the highest prevalence of *Mycoplasma* spp. (up to 100.00%) and *Ehrlichia* spp. (up to 35.71%) (Table 4). Noteworthy is the absence of *Rickettsia* spp., i.e., one of the most frequently detected pathogens in adult *I. ricinus* specimens (Table 2). This may suggest a limited role of *A. agrarius* and *M. glareolus* rodents as reservoirs of this pathogen [74].

## 5. Conclusions

In conclusion, two species of ixodid ticks, i.e., *Ixodes ricinus* and *Dermacentor reticulatus*, were found in the Western Carpathians and their foothills. *I. ricinus* is the dominant species, with a compact occurrence range, while *D. reticulatus* exhibits a more scattered distribution and limited population size in the region. Notably, the abundance of *I. ricinus* nymphs varies depending on the altitude.

The activity of *I. ricinus* ticks is significantly influenced by air temperature. Specimens of this species exhibit a circadian rhythm of questing activity. In the mountain habitats of the Western Carpathians, *Myodes glareolus* and *Apodemus agrarius* rodents serve as the main hosts for tick juvenile stages, while *Capreolus capreolus* roe deer can be considered preferred hosts for adult stages.

Two essential findings were that *I. ricinus* ticks act as crucial vectors and *A. agrarius* and *M. glareolus* as reservoirs of TBPs in the studied region.

## Figures and Tables

**Table 1:** Host range of ixodid ticks collected in studied area. L—larvae, N—nymphs, F—females, M—males.

Habitat Type	Host Species	Number of Tested Animals	Number of Infested Animals	Tick Species/Number of Collected Ticks								Prevalence (%)	Mean Tick Infestation per Animal
				*Ixodes ricinus*				*Dermacentor reticulatus*					
				L	N	F	M	L	N	F	M		
Forest	*Myodes glareolus*	18	16	30	49	0	0	0	0	0	0	88.9	4.9
	*Apodemus flavicollis*	3	1	0	0	0	0	0	6	0	0	33.3	6.0
	*Microtus* spp.	2	1	0	6	0	0	0	0	0	0	50.0	6.0
Meadow	*Apodemus agrarius*	24	14	6	36	0	0	0	6	0	0	58.3	3.4
	*Microtus* spp.	2	1	0	0	0	0	0	7	0	0	50.0	7.0
Open range	*Capreolus capreolus*	7	7	0	0	43	5	0	0	0	5	100.0	7.5
Pasture	*Ovis aries*	75	5	0	0	17	0	0	0	0	0	6.7	3.4

**Table 2:** Prevalence of tick-borne pathogens in *Ixodes ricinus* ticks collected in the studied area. * Minimum infection rate (*MIR* %) was calculated for pooled samples; *n*—number of tested samples, *pn*—number of pools.

Tick Collection Sites	Tick Stages	Tick-Borne Pathogens/Number of Positive Samples and Percentage Rate (%)												
		*Borrelia* spp.	*Borrelia burgdorferi* s.s	*Borrelia garinii*	*Borrelia afzelii*	*Borrelia valaisiana*	*Borrelia lusitaniae*	*Borrelia spielmanii*	*Borrelia miyamotoi*	*Anaplasma phagocytophilum*	*Ehrlichia* spp.	*Rickettsia* spp.	*Rickettsia helvetica*	*Babesia venatorum*
Meadow habitat of the Western Carpathians	Females *n* = 42	4 (9.52)	1 (2.38)	9 (21.42)	4 (9.52)	2 (4.76)	0 (0.00)	1 (2.38)	1 (2.38)	0 (0.00)	3 (7.14)	0 (0.00)	4 (9.52)	0 (0.00)
	Males *n* = 14	1 (7.14)	0 (0.00)	1 (7.14)	4 (28.57)	0 (0.00)	0 (0.00)	0 (0.00)	1 (7.14)	0 (0.00)	1 (7.14)	0 (0.00)	4 (28.57)	0 (0.00)
	Nymphs *pn* = 4	0 (0.00)	0 (0.00)	0 (0.00)	1 * (4.17)	0 (0.00)	0 (0.00)	0 (0.00)	0 (0.00)	0 (0.00)	0 (0.00)	0 (0.00)	1 * (4.17)	0 (0.00)
Forest habitat of the Western Carpathians	Females *n* = 42	1 (2.38)	0 (0.00)	6 (14.29)	2 (4.76)	0 (0.00)	4 (9.52)	0 (0.00)	0 (0.00)	1 (2.38)	2 (4.76)	0 (0.00)	9 (21.43)	1 (2.38)
	Males *n* = 14	1 (7.14)	0 (0.00)	1 (7.14)	1 (7.14)	0 (0.00)	1 (7.14)	0 (0.00)	0 (0.00)	0 (0.00)	1 (7.14)	1 (7.14)	0 (0.00)	0 (0.00)
	Nymphs *pn* = 4	0 (0.00)	0 (0.00)	1 * (4.17)	0 (0.00)	0 (0.00)	0 (0.00)	0 (0.00)	0 (0.00)	0 (0.00)	0 (0.00)	0 (0.00)	0 (0.00)	0 (0.00)
Forest habitat of the foothills of the Western Carpathians	Females *n* = 42	1 (2.38)	2 (4.76)	7 (16.67)	5 (11.90)	1 (2.38)	0 (0.00)	0 (0.00)	0 (0.00)	0 (0.00)	4 (9.52)	0 (0.00)	10 (23.81)	1 (2.38)
	Males *n* = 14	0 (0.00)	0 (0.00)	1 (7.14)	3 (21.43)	0 (0.00)	0 (0.00)	2 (14.29)	0 (0.00)	0 (0.00)	0 (0.00)	0 (0.00)	5 (35.71)	0 (0.00)
	Nymphs *pn* = 4	0 (0.00)	0 (0.00)	0 (0.00)	0 (0.00)	0 (0.00)	0 (0.00)	0 (0.00)	0 (0.00)	0 (0.00)	0 (0.00)	0 (0.00)	0 (0.00)	0 (0.00)

**Table 3:** Minimum infection rate (*MIR* %) of pathogens in juvenile ticks collected from rodents, *n*—number of tested pools (each pool consisted of 6 specimens).

Habitat Type	Rodent Species	Tick Species	Tick Stage	Tick-Borne Pathogens/Minimum Infection Rate (%)						
				*Borrelia* spp.	*Borrelia afzelii*	*Borrelia miyamotoi*	*Rickettsia* spp.	*Rickettsia helvetica*	*Bartonella* spp.	*Mycoplasma* spp.
Forest	*Myodes glareolus*	*Ixodes ricinus*	Larvae *n* = 5	0.00	0.00	0.00	10.00	0.00	10.00	0.00
			Nymphs *n* = 8	0.00	0.00	2.08	4.17	4.17	2.08	6.25
	*Microtus* spp.	*Ixodes ricinus*	Nymphs *n* = 1	0.00	0.00	0.00	0.00	0.00	0.00	16.67
	*Apodemus flavicollis*	*Dermacentor reticulatus*	Nymphs *n* = 1	0.00	0.00	0.00	0.00	0.00	0.00	0.00
Meadow	*Apodemus agrarius*	*Ixodes ricinus*	Larvae *n* = 1	0.00	0.00	0.00	0.00	16.67	0.00	0.00
			Nymphs *n* = 10	3.33	1.67	0.00	6.67	3.33	1.67	8.33
		*Dermacentor reticulatus*	Nymphs *n* = 1	0.00	0.00	0.00	0.00	16.67	0.00	0.00
	*Microtus* spp.	*Dermacentor reticulatus*	Nymphs *n* = 1	0.00	0.00	0.00	16.67	0.00	0.00	0.00

**Table 4:** Prevalence of pathogens and co-infections detected in the blood samples of the most common rodent species in each investigated habitat, *n*—number of tested samples.

Vector-Borne Pathogens	Forest Biotope	Meadow Biotope
	*Myodes glareolus* *n* = 16	*Apodemus agrarius* *n* = 14
	Number of Positive Samples and Percentage Rate (%)	
*Bartonella* spp.	4 (25.00)	0 (0.00)
*Mycoplasma* spp.	6 (37.50)	7 (50.00)
*Borrelia* spp. + *Mycoplasma* spp.	1 (6.25)	0 (0.00)
*Bartonella* spp. + *Mycoplasma* spp.	3 (18.75)	2 (14.29)
*Ehrlichia* spp. + *Mycoplasma* spp.	0 (0.00)	4 (28.57)
*Ehrlichia* spp. + *Bartonella* spp. + *Mycoplasma* spp.	0 (0.00)	1 (7.14)

## Data Availability

The datasets generated during and/or analyzed during the current study are available from the corresponding authors upon reasonable request.

## Supplementary Materials

The following supporting information can be downloaded at: https://www.mdpi.com/article/10.3390/pathogens12091186/s1, Table S1. Total number of *Ixodes ricinus* ticks collected at particular study sites during whole study period, depending on the latitude; m a.s.l.—meters above sea level, N—nymphs, F—females, M—males. Table S2. Co-infections of pathogens detected in questing *I. ricinus* adults collected in studied habitats, n—number of tested samples.
