# Supplementary material for: Tick Activity, Host Range, and Tick-Borne Pathogen Prevalence in Mountain Habitats of the Western Carpathians, Poland
Source: Pathogens. 2023 Sep 21;12(9):1186. doi: 10.3390/pathogens12091186 (PMC10534405; doi:10.3390/pathogens12091186)
Supplement: Supplementary file 1 [file pathogens-12-01186-s001.zip › Table S1.pdf]

Table S1.Total number of *Ixodes ricinus* ticks collected at particular study sites during whole study period, depending on the latitude; m a.s.l. – meters above sea level, N – nymphs, F – females, M – males.

**Studied region**	**Study site**	**Latitude (m a.s.l)**	**Tick stage/Total number of collected ticks**			**Total**
			**N**	**F**	**M**	
Foothill of Western Carpathians mountains	A (Rzeszów N)	191	62	59	57	178
	B (Rzeszów S)	251	40	53	45	138
	C (Jawornik)	284	12	70	54	136
	D (Kombornia)	363	30	65	50	145
	E (Rogi)	299	109	85	87	281
Western Carpathians mountains	F (Chyrowa)	529	94	74	79	247
	G (Ropianka)	465	115	75	77	267
	H (Mszana)	447	94	98	71	263
	J (Barwinek)	470	209	76	68	353
Total			765	655	588	2008
