# Supplementary material for: Tick Activity, Host Range, and Tick-Borne Pathogen Prevalence in Mountain Habitats of the Western Carpathians, Poland
Source: Pathogens. 2023 Sep 21;12(9):1186. doi: 10.3390/pathogens12091186 (PMC10534405; doi:10.3390/pathogens12091186)
Supplement: Supplementary file 1 [file pathogens-12-01186-s001.zip › Table S2.pdf]

Table S2. Co-infections of pathogens detected in questing *I. ricinus* adults collected in studied habitats, n – number of tested samples.

Co-infections	Number of positive samples and percentage rate (%)					
	Meadow habitat of the Western Carpathians		Forest habitat of the Western Carpathians		Forest habitat of the foothills of the Western Carpathians	
	Females n = 42	Males n = 14	Females n = 42	Males n = 14	Females n = 42	Males n = 14
Borrelia valaisiana + *Borrelia miyamotoi*	1 (2.38)	0 (0.00)	0 (0.00)	0 (0.00)	0 (0.00)	0 (0.00)
Borrelia afzelii + *Rickettsia helvetica*	0 (0.00)	1 (7.14)	0 (0.00)	0 (0.00)	0 (0.00)	0 (0.00)
Borrelia spp. + *Ehrlichia* spp.	1 (2.38)	0 (0.00)	0 (0.00)	0 (0.00)	0 (0.00)	0 (0.00)
Rickettsia helvetica + *Ehrlichia* spp.	0 (0.00)	1 (7.14)	1 (2.38)	0 (0.00)	1 (2.38)	0 (0.00)
Borrelia lusitaniae + *Babesia venatorum*	0 (0.00)	0 (0.00)	2 (4.76)	0 (0.00)	0 (0.00)	0 (0.00)
Borrelia afzelii + *Ehrlichia* spp.	0 (0.00)	0 (0.00)	1 (2.38)	0 (0.00)	2 (4.76)	0 (0.00)
Borrelia lusitaniae + *Rickettsia* spp.	0 (0.00)	0 (0.00)	0 (0.00)	1 (7.14)	0 (0.00)	0 (0.00)
